# Multifunctional Hybrid Material for Endoprosthetic Implants Based on Alumina-Toughened Zirconia Ceramics and Additively Manufactured TiNbTa Alloys

**DOI:** 10.3390/ma17081838

**Published:** 2024-04-16

**Authors:** Jan-Oliver Sass, Paul Henke, Aurica Mitrovic, Markus Weinmann, Daniel Kluess, Jan Johannsen, Marie-Luise Sellin, Ulrich Lembke, Daniel Reimer, Cornelia Lork, Anika Jonitz-Heincke, Rainer Bader

**Affiliations:** 1 Research Laboratory for Biomechanics and Implant Technology, Department of Orthopaedics, Rostock University Medical Center, Doberaner Straße 142, D-18057 Rostock, Germany; paul.henke@med.uni-rostock.de (P.H.);; 2 ZM Praezisionsdentaltechnik GmbH, Breite Str. 16, D-18057 Rostock, Germanycornelia.lork@zm-dental.de (C.L.); 3 TANIOBIS GmbH, Im Schleeke 78-91, D-38642 Goslar, Germany; markus.weinmann@taniobis.com; 4 INNOPROFF GmbH, Joachim-Jungius-Straße 9, D-18059 Rostock, Germany; 5 Fraunhofer Research Institution for Additive Manufacturing Technologies IAPT, Am Schleusengraben 14, D-21029 Hamburg, Germany; jan.johannsen@iapt.fraunhofer.de; 6 DOT GmbH, Charles-Darwin-Ring 1A, D-18059 Rostock, Germany; 7 FMZ GmbH, Charles-Darwin-Ring 3A, D-18059 Rostock, Germany

**Keywords:** joint arthroplasty, total knee replacement, implant, aseptic loosening, material joining, alumina-toughened zirconia, beta titanium, glass soldering, additive manufacturing

## Abstract

Aseptic implant loosening after a total joint replacement is partially influenced by material-specific factors when cobalt–chromium alloys are used, including osteolysis induced by wear and corrosion products and stress shielding. Here, we aim to characterize a hybrid material consisting of alumina-toughened zirconia (ATZ) ceramics and additively manufactured Ti-35Nb-6Ta (TiNbTa) alloys, which are joined by a glass solder. The structure of the joint, the static and fatigue shear strength, the influence of accelerated aging, and the cytotoxicity with human osteoblasts are characterized. Furthermore, the biomechanical properties of the functional demonstrators of a femoral component for total knee replacements are evaluated. The TiNbTa-ATZ specimens showed a homogenous joint with statistically distributed micro-pores and a slight accumulation of Al-rich compounds at the glass solder–TiNbTa interface. Shear strengths of 26.4 ± 4.2 MPa and 38.2 ± 14.4 MPa were achieved for the TiNbTa-ATZ and Ti-ATZ specimens, respectively, and they were not significantly affected by the titanium material used, nor by accelerated aging (*p* = 0.07). All of the specimens survived 10^7^ cycles of shear loading to 10 MPa. Furthermore, the TiNbTa-ATZ did not impair the proliferation and metabolic activity of the human osteoblasts. Functional demonstrators made of TiNbTa-ATZ provided a maximum bearable extension–flexion moment of 40.7 ± 2.2 Nm. The biomechanical and biological properties of TiNbTa-ATZ demonstrate potential applications for endoprosthetic implants.

## 1. Introduction

Aseptic implant loosening is the main reason for the revision of total joint replacements [1]. Implant-material-related complications are associated with wear particles, corrosion products, and the mechanical mismatch of the materials to the human bone [2,3]. Metal wear particles can stimulate osteoclastic bone resorption [2] and released metal ions, e.g., from cobalt–chromium and titanium alloys (Co^2+^, Cr^3+^, Al^3+^, and V^2+^), may cause adverse local [4,5,6] and systematic biological responses [7,8,9,10,11]. Commonly used implant materials lead to the mechanical loading alteration of the periprosthetic bone (i.e., stress shielding). The mechanical stimulus on the bone tissue and cells is reduced; thus, the bone remodeling is shifted toward resorption [2,3]. These effects lead to periprosthetic bone loss, thereby potentially causing osteolysis, implant loosening, and increased periprosthetic fracture risk [12].

Multifunctional hybrid implant materials have been investigated to address these issues [13,14,15,16,17,18,19,20,21]. These materials are supposed to combine the advantageous properties of oxide ceramics at the articulating surfaces of the artificial joint and titanium (Ti) alloys at the bone–implant interface. Thus, high wear and corrosion resistance with a lower risk of stress shielding and improved osseointegration can be achieved [13,15,17,22]. In previous studies, functionally graded materials manufactured by spark plasma sintering have been investigated [13,14,15,16,17,22]. They are composed of a pure ceramic phase (e.g., Al_2_O_3_ or Y_2_O_3_-stabilized ZrO_2_), graded ceramic–titanium phases with continuously decreasing ceramic content, and a titanium or Ti-6Al-4V phase. Other approaches have used laser-engineered net shaping to manufacture Ti6Al4V-Al_2_O_3_ [18] hybrids or glass solders to join solid ceramic- and titanium-based components [20,21].

The glass soldering of bioceramics such as Al_2_O_3_ or ZrO_2_ and commercially pure titanium (cp-Ti) was originally intended for dental applications [23], but it has since demonstrated applicability to endoprosthetic implant materials like alumina-toughened zirconia (ATZ) ceramics and Ti-6Al-4V [21]. Processing such hybrid materials involves the application of a biocompatible silica-based glass [21,24] to the joining surfaces, as well as a subsequent firing to melt the glass solder. The main reasons for a stable and durable connection are the formation of reaction layers during a firing that is comparable to other metal–ceramic composites [20,25], and the mechanical interlocking between glass solder and the ceramic or the metal part [20]. For example, Mick et al. [21] used a glass solder (main components: SiO_2_, Al_2_O_3_, Na_2_O, and KO_2_) to fabricate Ti6Al4V-ATZ hybrid materials, and they reported a bending strength of 118 ± 33 MPa. In addition, Markhoff et al. [26] showed a good interaction of human osteoblasts with a similar glass solder that was applied as a coating on ATZ bulk material. Nevertheless, the transformation of this technology to endoprosthetic implants, such as the femoral component of a total knee replacement, presents challenges in joining larger- and complex-shaped surfaces.

While the number of ceramic systems for endoprosthetic implant applications is limited so far, a variety of metallic implant materials is available. In this regard, Ti and its alloys are state-of-the-art materials that feature good biocompatibility, i.e., cell tolerance and osseointegration [27,28,29,30]. Standard titanium materials are commercially pure Ti (cp-Ti) and Ti-6Al-4V [27,30], where the latter provides high survival rates for endoprosthetic implants [31]. However, both suffer from their stiffness, which is expressed by a high elastic modulus of ~110 GPa [27], which thus poses a major stress shielding risk [32,33]. It has also been discussed that released aluminum and vanadium ions potentially cause adverse biological effects in the organism [9,11,34].

Consequently, extensive studies have been performed to explore advanced biocompatible Ti-based alloys, featuring a favorable combination of high elasticity (a low Young’s modulus), high strength, good fatigue properties, and biocompatibility. Hence, binary, ternary, quaternary, and even multi-component high-entropy Ti base alloys have been developed for use as metallic implant materials [35,36,37,38,39,40,41,42,43,44,45,46,47,48,49,50,51].

The most thoroughly investigated binary Ti alloy systems are Ti/Nb [35,36,37,38] and Ti/Ta [39,40,41,52]. Their phase composition and mechanical properties depend on the Ti:(Nb/Ta) ratio. Ti-rich alloys predominantly crystallize hexagonally with an α or α’ crystal structure. Both Nb and Ta are so-called β stabilizers and, accordingly, Nb or Ta-rich alloys crystallize in the cubic-body-centered β structure [53,54]. Moreover, an orthorhombic α’’ crystal structure and α + β compositions were reported. The mechanical properties of α and β can be quite different: alloys possessing the α phase are usually stronger, whereas those with β structure are more elastic, thereby providing better mechanical compatibility with the cortical and trabecular bone [27,28,55].

Combining Ti with both Nb and Ta leads to ternary Ti/Nb/Ta alloys. They have also attracted scientific attention, and the diversity of materials and compositions is much higher than in binary subsystems. Some alloy compositions have even been proven to exhibit a shape memory effect or superelasticity [43,46]. Typical Ti/Nb/Ta alloys that have been investigated for biomedical applications are, e.g., β-phase Ti-25Nb-25Ta, which has been processed by cold crucible levitation melting [42]. This alloy revealed a low Young’s modulus of about 55 GPa, as well as good ductility and strength, i.e., 20% elongation at fracture and about 530 MPa ultimate tensile strength. The Ti-30Nb-18Ta, which was arc-melted, solution-treated, and then 50% cold-rolled, predominantly consisted of the orthorhombic α″ martensitic phase embedded in a β-phase matrix [44]. An important finding was the efficient passivation of Ti-30Nb-18Ta due to the presence of Nb and Ta, which form chemically inert native oxide surfaces that protect the alloys from the further oxidation of, e.g., body fluids.

Recently, Ti/Nb/Ta alloys in the Ti-rich domain were described. Materials with a Ti-xNb-6Ta (x = 20, 27, and 35) chemical composition were especially developed for application in additive manufacturing processes [45]. The goal was to produce patient-specific dental and orthopedic implants with the highest level of biocompatibility using laser beam powder bed fusion (PBF-LB/M). It turned out that the compressive modulus could be lowered to ~43 GPa in the case of Ti-20Nb-6Ta [47]. This is in the range of the most elastic Ti/Nb/Ta/Zr (TNTZ) [46,48,49,51,56] alloys, which are also referred to as gum metal [50] due to their high elasticity, or to Ti/Nb/Zr/Sn (TNZS), which possess similar characteristics [57,58]. However, the chemistry and controllability across the entire process chain is significantly simpler for ternary Ti/Nb/Ta alloys compared to TNTZ, which is an important factor with regard to commercial applications.

The present study aims to characterize hybrid TiNbTa-ATZ specimens using additively manufactured Ti-35Nb-6Ta components that are joined to ATZ using a biocompatible silica-based glass solder. The manufactured TiNbTa-ATZ joints were structurally and chemically analyzed by backscatter electron (BSE) microscopy and energy-dispersive X-ray spectroscopy (EDX). Furthermore, the hybrid material was characterized by mechanical testing (i.e., of static and fatigue shear stress), and the influence of artificially aging on static shear strength was analyzed. Hybrid Ti-ATZ specimens were used as a reference. In addition, the cytotoxicity of TiNbTa-ATZ specimens was evaluated and compared with Ti-ATZ and Co-28Cr-6Mo specimens using an elution assay and human osteoblasts. Furthermore, a simplified functional implant demonstrator of a hybrid material-based femoral component was fabricated, structurally characterized, and analyzed for its mechanical strength under biomechanical loading using gait cycles, as well as loading to failure.

## 2. Materials and Methods

### 2.1. Manufacturing of the Hybrid Material

The hybrid material specimens made of slip-casted ATZ (Koebel Engineering, Dachsen, Switzerland), and the TiNbTa were manufactured and joined by applying glass soldering. Hybrid Ti-ATZ materials were used as a reference since glass soldering was initially developed to join cp-Ti and zirconia-based oxide ceramics in the field of dentistry [23]. The TiNbTa components were additively manufactured using PBF-LB/M. The spherical TiNbTa powder was produced by electrode induction melting gas atomization (EIGA), which was conducted under a purified argon (4.6, Linde GmbH, Pullach, Germany) atmosphere from pre-alloyed electrodes (TANIOBIS GmbH, Goslar, Germany) [45]. In a previous study with similar powder, the measured chemical composition, which was determined by inductively coupled plasma optical emission spectroscopy (ICP-OES) was 58.87 wt. % Ti, 34.45 wt. % Nb, and 5.98 wt. % Ta [45]. PBF-LB/M was performed using a DMP350 Flex (3D Systems Corp., Rock Hill, SC, USA) equipped with a 1 kW single-mode laser (YLR-1000-WC-Y14, IPG Laser GmbH, Burbach, Germany) under an argon gas atmosphere to prevent oxidation. The scanning speed was 1500 mm × s^−1^, the laser power was 170 W, the layer thickness was 0.3 mm, and the hatch distance was 69 µm. Similar powder, devices, and process parameters led to dense parts with a homogenous element distribution and a monocrystalline β-phase [45]. After additive manufacturing, all TiNbTa components were heat-treated for 4 h at 1200 °C in a vacuum. Within the preliminary tests, we observed that this was necessary to reduce the residual stresses during glass soldering. Furthermore, the end faces of the cylindrical TiNbTa specimens used for shear testing were machined to meet the parallelism requirements of the joining surfaces.

The main components of the silica-based glass solder (DCMhotbond fusio-12, DCM Dental Creative Management GmbH, Rostock, Germany) were SiO_2_ (63–67 wt. %), Al_2_O_3_ (6–9 wt. %), K_2_O (6–9 wt. %), and Na_2_O (6–9 wt. %). The glass solder had a coefficient of thermal expansion (CTE) of 10.0 × 10^−6^ K^−1^, a melting temperature of 450 °C, and a bending strength at room temperature of ≥50 MP (provided by ZM Praezisionsdentaltechnik GmbH, Rostock, Germany). The ATZ ceramics (provided by Koebel Engineering, Dachsen, Switzerland) had a CTE of 7.8–8.1 × 10^−6^ K^−1^. Further, the CTE of Ti-35Nb-6Ta (provided by TANIOBIS GmbH, Goslar, Germany) was temperature-dependent and ranged, in the relevant temperature regime of 20 °C to 450 °C (i.e., the melting point of the glass solder), from 8.2 × 10^−6^ K^−1^ to 9.3 × 10^−6^ K^−1^. Similar to previous studies [21,24], the soldering was performed in a furnace and according to the guidelines provided by DCM Dental Creative Management GmbH, Rostock, Germany. Before glass soldering, the joining surfaces were sandblasted (110 µm Al_2_O_3_ at 4 bar), cleaned in an ultrasonic bath in ethanol, and then primed with a thin layer of the glass solder. After sandblasting, the TiNbTa and cp-Ti components had an average roughness (measured using a VK-X250 laser scanning microscope, Keyence Corporation, Osaka, Japan) of 1.5 ± 0.1 µm and 1.7 ± 0.1 µm, respectively. Finally, a glass solder paste was applied to the joining surfaces and fired at 820 °C for 5 min in a vacuum using a heating and cooling rate of 20 K·min^−1^. The joined interface of the TiNbTa-ATZ specimens was analyzed by BSE and EDX using an SEM JSM6490 (Jeol, Akishima, Tokyo, Japan) equipped with an X-Flash SEM 4010 (Bruker Nano GmbH, Berlin, Germany) for structural and chemical analysis.

### 2.2. Shear Testing, Artificial Aging, and Fracture Analysis

**Figure 1:**
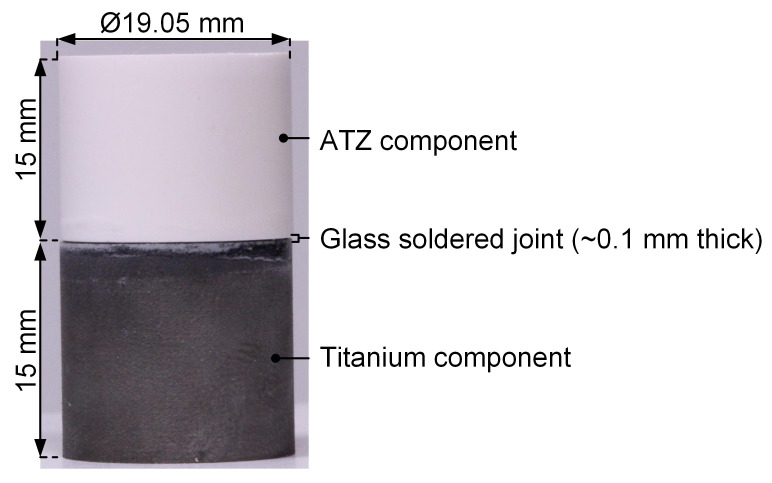
The shear specimens were based on hybrid materials consisting of an alumina-toughened zirconia (ATZ) ceramic and an additively manufactured Ti-35Nb-6Ta (TiNbTa) or a cp-Ti joined with a silica-based glass solder.

The static and dynamic shear testing of the hybrid materials was performed according to the relevant standards [59,60,61]. The dimensions of the shear test specimens are shown in Figure 1.

Furthermore, the influence of artificial aging (0.5 MPa, 70 °C, 14 days [62]) on the static shear strength was evaluated. Accordingly, six different groups (Table 1) were characterized, and each group contained *n* = 5 specimens.

The static shear tests were conducted using a universal testing machine (Zwick 50kN RetroLine, Zwick Roell, Ulm, Germany). The specimens were loaded until fracture at a rate of 2.5 mm × min^−1^. The fatigue tests were performed using an electro-dynamic testing machine (ElectroForce 3510, TA Instruments—Waters LLC, Eden Prairie, MN, USA) with a sinusoidal load between 1 MPa and 10 MPa and a frequency of 10 Hz [63]. Furthermore, 10^7^ cycles were defined as a successful test [59].

After mechanical testing, the fractured surfaces of all specimens were analyzed with a digital microscope (VHX-6000) and laser scanning microscope (VK-X250) (both obtained from Keyence Corporation, Osaka, Japan) to determine the causes of fracture.

### 2.3. Biological Characterization

For the biological characterization of the hybrid material specimens (TiNbTa-ATZ and Ti-ATZ), cytotoxicity measurements were performed by an elution assay, and commercially used Co-28Cr-6Mo specimens served as the negative control. The specimens had a diameter of 12 mm and were 5 mm in height. The heat-sterilized specimens were first covered with 838 µL of calcium-free Dulbecco’s Modified Eagle’s Medium (DMEM) per sample, and they were incubated at 37 °C and 5% CO_2_ for 14 and 21 days. A medium control without samples was included. The medium eluates were stored at −20 °C until use.

For the cytotoxicity assays, human osteoblasts were isolated from the femoral heads of the patients undergoing total hip arthroplasty according to an established protocol by Lochner et al. [64]. Femoral heads were provided after informed consent was obtained from the patients. The study was approved by the ethics committee of the University Medical Center Rostock (A 2010-0010). Experiments were performed with human osteoblasts from a total of six donors (n = 8, female: n = 6, mean age: 61 ± 7.3 years; male: n = 2, mean age: 53.5 ± 3.5 years). Cells were cultured under standard culture conditions at 37 °C and 5% CO_2_ in a calcium-free DMEM supplemented with 10% fetal calf serum (FCS; both: PAN-Biotech, Aidenbach, Germany), 1% amphotericin B, 1% penicillin-streptomycin, and 1% HEPES buffer (all: Sigma-Aldrich, Munich, Germany). To maintain the osteogenic phenotype, 10 mM of β-glycerophosphate, 50 μg × mL^−1^ of ascorbic acid, and 100 nM of dexamethasone were added to the cell culture medium (all: Sigma-Aldrich, Munich, Germany). Moreover, 10,000 osteoblasts per well were seeded in a 96-well plate (Thermo Fisher Scientific Inc., Waltham, MA, USA).

The eluates of the hybrid materials, the Co-28Cr-6Mo specimens, and controls were thawed and diluted 1:1 with a fresh medium containing osteogenic additives to use them for the exposure of the osteoblastic cells. Osteoblasts were incubated with 150 µL of the diluted eluate for 24 h. Afterward, the viability of osteoblasts after incubation was evaluated via the metabolic activity assay water-soluble tetrazolium salt (WST-1; Takara Bio, Saint-Germain-en-Laye, France) and the CyQUANT™ NF Cell Proliferation Assay (ThermoFisher Scientific, Waltham, MA, USA). First, the metabolic activity was determined. Then, the diluted eluates were removed and the cells were washed with PBS. The cells were then incubated with a defined volume of WST-1/medium reagent (1:10 ratio) at 37 °C and 5% CO_2_. After an incubation period of 30 min, 100 µL of the supernatants were transferred to a 96-well cell culture plate, and the absorbance at 450 nm (reference wavelength: 630 nm) was measured in a microplate reader (Tecan Reader Infinite^®^ 200 Pro, Tecan Trading AG, Maennedorf, Switzerland). To determine the absolute cell number, the CyQUANT™ Cell Proliferation Assay was performed according to the manufacturer’s guidelines. The same cells for which the metabolic activity was previously determined were used. Cells were covered with 100 µL of 1× Dye Binding Solution (consisting of 1:500 Dye Reagent and 1× HBSS), incubated at 37 °C, and protected from light. After 60 min, the fluorescence intensity was measured at 530 nm (excitation wavelength: 485 nm) using the Tecan Infinite^®^ 200 Pro reader. A cell number calibration curve was generated using pre-defined cell numbers in duplicate to relate the fluorescence signal to the actual cell number.

### 2.4. Biomechanical Characterization of Functional Demonstrators

#### 2.4.1. Demonstrator Manufacturing

**Figure 2:**
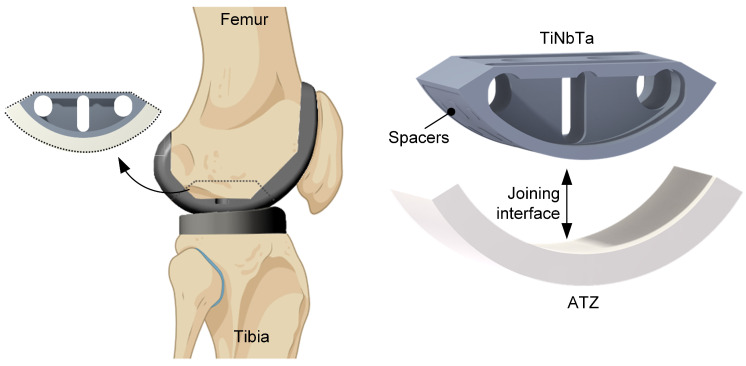
Design of the simplified functional demonstrator of a hybrid-material-based femoral component for a total knee replacement resembling a part of the tibiofemoral joint. The hybrid material is formed by a glass soldering of the additively manufactured TiNbTa to ATZ ceramics, and the joining surface of TiNbTa is functionalized with spacers that are 0.1 mm in height to ensure a homogeneous joint gap (created with Biorender.com).

Based on the geometries of commercially available total knee endoprostheses [65], a simplified functional demonstrator was designed in Creo Parametrics 10.0.0.0 (PTC, Boston, MA, USA) to gain experience with more complex-shaped soldering components. The simplification was necessary to achieve the next development step. The functional demonstrator represented one condyle of the tibiofemoral joint (Figure 2) with an outer radius of 30 mm and a depth of 22.5 mm. Since a homogeneous joint gap is crucial for the glass soldering process, a frame and spacers were designed on the titanium component with a height of 0.1 mm. The titanium-based component was further designed to enable clamping of the functional demonstrator during the biomechanical testing, and geometric cutouts were designed to reduce the amount of heat absorption during the firing process. A detailed depiction of the dimensions is shown in Appendix A.

**Figure 3:**
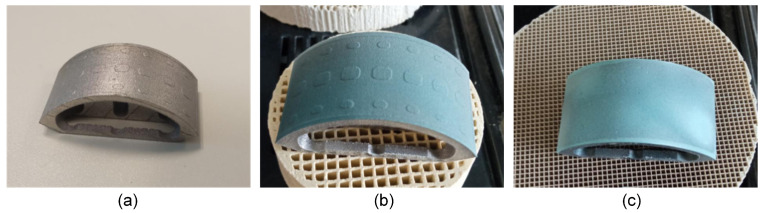
The consecutive steps taken to prime the Ti-based component (TiNbTa or cp-Ti) of the hybrid-material-based functional demonstrator with (**a**) an untreated specimen, (**b**) a specimen coated with the glass solder, and (**c**) a completely primed specimen by stepwise firing and polishing the glass solder to fill the gap between the designed spacers with the glass solder. The glass solder was dyed blue for better visualization.

For glass soldering, the joint surfaces were sandblasted (110 µm Al_2_O_3_ at 4 bar) and cleaned with ethanol in an ultrasonic bath for 3 min. The average roughness values (measured by laser scanning microscopy) of the sandblasted TiNbTa (PBF-LB/M) and cp-Ti (CNC machined) components were 5.8 ± 2.0 µm and 1.9 ± 0.3 µm, respectively. In general, the soldering followed the same procedure as described in Section 2.1, but a more extensive priming of the surfaces was conducted to omit the pore formation in the soldered joint. The first priming was performed by spray coating with the glass solder paste. A firing process of the individual parts was subsequently conducted to, respectively, establish the joint between the glass solder, the ATZ, and the titanium-based material. After the first priming of the titanium-based component, the soldering surface was polished (600 µm and 1000 µm grit), covered with glass solder paste, and then fired again. This process was repeated two times to fill the room between the spacers with the glass solder. In Figure 3, the initial additively manufactured TiNbTa component along with the spray-coated specimen and the final stage of priming are illustrated.

The soldering of the ATZ and the TiNbTa or cp-Ti component was conducted at 820 °C for 5 min in a vacuum. The structure quality of the joint interface was analyzed by conducting an electron microscopy of a polished cross-section of the hybrid TiNbTa-ATZ specimen.

#### 2.4.2. Biomechanical Characterization

The biomechanical characterization of the functional demonstrators comprised two consecutive tests. First, the specimens were loaded for 10,000 walking cycles; second, the same specimens were used to evaluate the maximum extension–flexion moment. Each group of either TiNbTa-ATZ or Ti-ATZ hybrids contained *n* = 3 specimens.

**Figure 4:**
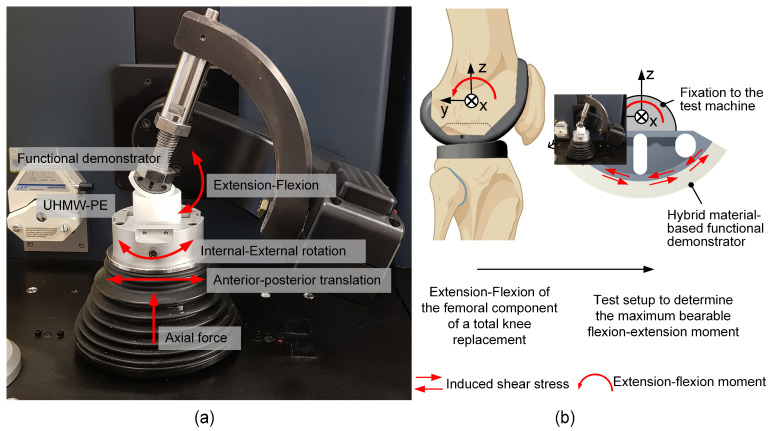
Biomechanical characterization of the hybrid-material-based (glass soldered TiNbTa-ATZ or Ti-ATZ) functional demonstrators of the femoral component of a total knee replacement: (**a**) biomechanical loading of the walking cycle in the VIVO^TM^ joint simulator and (**b**) schematic illustration of the flexion movement of the tibiofemoral joint and the derived test setup to characterize the maximum bearable extension–flexion moment (created with Biorender.com).

The 10,000 walking cycles were applied using a 6-degree-of-freedom joint simulator (VIVO^TM^, Advanced Mechanical Technology, Watertown, MA, USA), which was used in accordance with ISO standard 14243-3:2014 [61]. Therefore, the extension–flexion, internal–external rotation, and anterior–posterior translation were position-controlled, and the superior–inferior direction was force-controlled (axial force). The abduction–adduction rotation and medial–lateral translation remained unloaded. The functional demonstrators were articulated with cylindrical ultra-high-molecular-weight polyethylene specimens with a flat surface. Silicone oil (Typ 350, Caesar & Loretz GmbH, Hilden, Germany) served as a lubricant. The test setup and applied rheonomic constraints are shown in Figure 4a. Moreover, the specimens that survived the dynamic loading were rotationally loaded until failure with 0.1 ° × s^−1^ (Figure 4b) to simulate an extension–flexion moment. The ATZ component was constrained in the yz-plane (sagittal plane), and the moment was applied through the titanium component and the rotational center of the functional demonstrator.

### 2.5. Statistical Analysis

Statistical analysis of the results was performed in GraphPad Prism 9.2 (GraphPad Software, San Diego, CA, USA), and *p* < 0.05 was used as the level of significance. The results of the shear testing were checked for significant differences using the Mann–Whitney U Test. For the cytotoxicity tests, comparisons between the experimental groups were performed using 2-way ANOVA and the Bonferroni multiple comparison test. All data are presented as individual values with median and interquartile ranges.

## 3. Results

### 3.1. Structural, Chemical, and Mechanical Characterization

**Figure 5:**
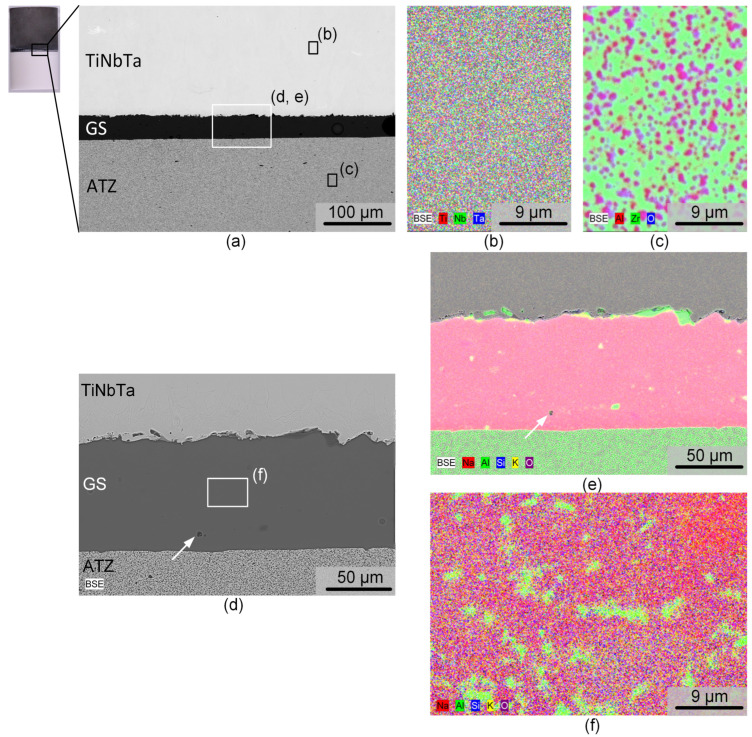
Electron microscopic images of a hybrid material of an alumina-toughened zirconia (ATZ) ceramic and additively manufactured Ti-35Nb-6Ta (TiNbTa) that were joined using a silica-based glass solder. (**a**,**d**) Backscatter electron microscopy of the investigated cross-section at different magnifications. (**b**,**c**,**e**,**f**) Element distribution in the TiNbTa alloy, (**b**) the ATZ ceramic, and (**c**) the glass solder (**e**,**f**). Pores are indicated by white arrows.

The structural and chemical analysis by electron microscopy of a polished cross-section of a TiNbTa-ATZ specimen is shown in Figure 5. The BSE image in Figure 5a points to the fact that the soldering occurred very homogeneously. The thickness of the solder was slightly below 100 µm. Occasionally, spherical pores were visible in the solder with diameters in the single-digit micro region, as evident from the magnified spot displayed in Figure 5d. The elemental mapping of the TiNbTa component (Figure 5b) displayed a homogenous element distribution of the constituting elements. In contrast to the as-atomized TiNbTa powder, no segregation in the Ti- or Nb/Ta-enriched dendrite-type structures was observed. The ATZ (Figure 5c) represents a two-phase material in which sub-micron Al_2_O_3_ particles were evenly embedded in a ZrO_2_ matrix. The element mapping of the solder displayed in Figure 5e reveals a local accumulation of aluminum at the glass solder–TiNbTa interface. Besides these features, the glass solder was constituted by a homogenous SiO_2_, K_2_O, and Na_2_O matrix containing Al_2_O_3_ segregations (Figure 5f).

**Figure 6:**
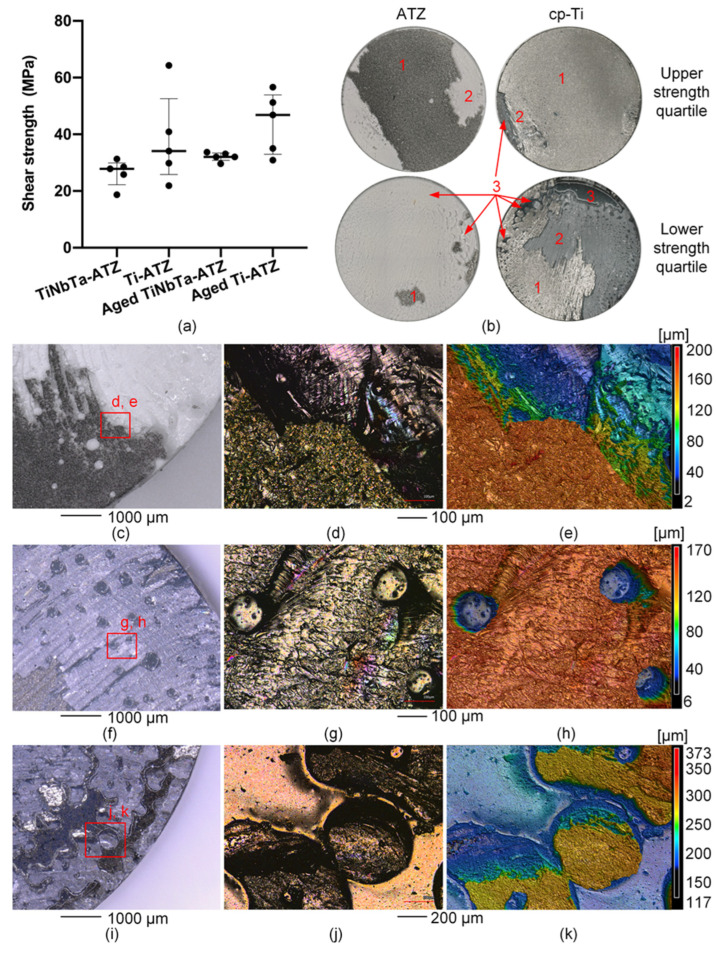
Results of the static shear testing and microscopy of the fracture surfaces of the glass-soldered TiNbTa-ATZ or Ti-ATZ hybrids. (**a**) The static shear strength (the data are presented as single values (indicated by bullets) with median and interquartile ranges). (**b**) The representative fracture surfaces of the specimens in the upper and lower quartile of the static strength (1: cohesive failure of the Ti-based component, 2: adhesive failure of the glass solder, and 3: imperfections in the glass solder due to spherical pores). (**c**–**e**) The microscopic images and depth profile of an ATZ fracture surface of a Ti-ATZ specimen indicating the cohesive failure of the cp-Ti, which led to the deposition of the bulk material on the ATZ surface. (**d**–**h**) The microscopic images and depth profile of a TiNbTa fracture surface of a Group 1 specimen indicating spherical pores in the glass solder. (**i**–**k**) The microscopic images and depth profile of an cp-Ti fracture surface of a Group 4 specimen indicating networked or branched structures in the glass solder.

The static shear strength between the tested groups was not statistically different (*p* = 0.07) (see Figure 6a), and all specimens showed a brittle fracture behavior. The representative stress–displacement curves are shown in Appendix B. The static shear strength of the TiNbTa-ATZ and Ti-ATZ specimens (Group 1 vs. Group 2) was 26.4 ± 4.2 MPa and 38.2 ± 14.4 Mpa, respectively. The static shear strength of the artificially aged TiNbTa-ATZ and Ti-ATZ specimens (Group 3 vs. Group 4) was 32.1 ± 1.4 Mpa and 44.1 ± 9.7 Mpa, respectively. Both hybrid materials (Groups 5 and 6) survived 10^7^ cycles at a 10 Mpa dynamic shear loading without fracture.

A mixed mode of failure in the Ti-based bulk material (cohesive failure) and failure in the glass solder (adhesive failure) was observed in different proportions (Figure 6b). Cohesive failure was visible as a deposition of the material on the ATZ component, as can be seen by the depth profile in Figure 6e. Furthermore, it was observed that specimens with a predominantly cohesive failure had higher shear strengths compared to the specimens that mainly fractured in the glass solder (Figure 6b). The pore formation locally hindered the bonding of the materials, thereby causing imperfections in the soldered joint. These imperfections were randomly distributed across the surfaces and were either spherical (Figure 6f–h) or formed a networked or branched structure (Figure 6i–k).

### 3.2. Biological Characterization

An eluate test was performed to indirectly determine the cytotoxicity of the hybrid materials. For this purpose, specimens of the hybrids and Co-28Cr-6Mo (the negative control) were incubated in osteoblastic cell culture medium over a period of 14 and 21 days. Afterward, the eluates were used for the cell experiments.

**Figure 7:**
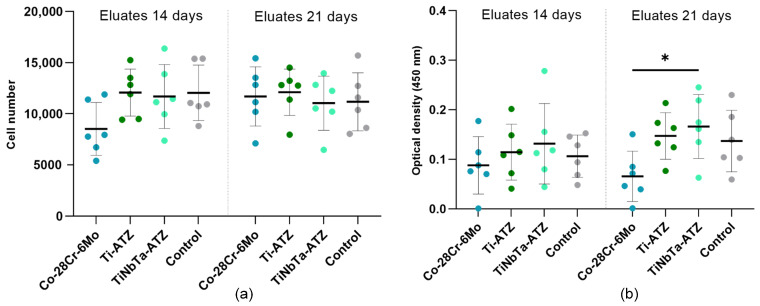
Cytotoxicity analysis of the glass-soldered, hybrid material specimens (TiNbTa-ATZ or Ti-ATZ) and Co-28Cr-6Mo (negative control) using elution testing. For this purpose, the specimens were incubated in a cell culture medium over 14 and 21 days. Afterward, the human osteoblasts were exposed to the eluates over a period of 24 h. Quantification of (**a**) the cell number by CyQUANT™ assay and (**b**) the metabolic activity by WST-1 assay. The osteoblasts in a cell culture medium served as the control. The data of six individual donors are presented as single values with median and interquartile ranges. Statistical significance was determined by two-way ANOVA followed by a Bonferroni multiple comparison test. * *p* < 0.05.

The eluates from the hybrid material specimens did not affect the proliferation of human osteoblasts (Figure 7a). No difference could be detected between the medium controls (dashed line) or between the incubation times of the eluates. However, a slight reduction in cell number was detected for the cells exposed to the 14-day eluates of the Co-28Cr-6Mo alloy.

The metabolic activity of osteoblasts was not influenced after exposure to the 14-day eluates (Figure 7b). In contrast, the incubation of cells with the 21-day eluates of the TiNbTa-ATZ specimens resulted in a significantly higher metabolic activity compared to those of Co-28Cr-6Mo (*p* = 0.031). Compared to the cells exposed to the 21-day negative control eluates, the eluates of the hybrid test specimens did not influence the metabolic activity.

### 3.3. Biomechanical Characterization of the Demonstrator

**Figure 8:**
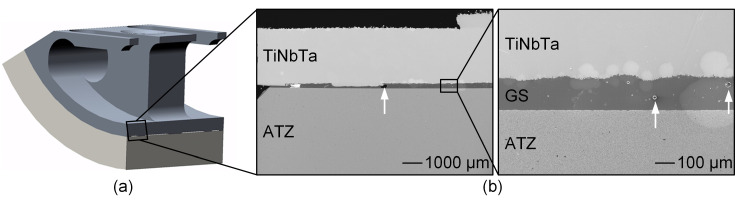
Structural analysis of the functional demonstrator of the femoral component of a total knee replacement made of alumina-toughened zirconia (ATZ) ceramic and additively manufactured Ti-35Nb-6Ta (TiNbTa) that were joined by a silica-based glass solder (GS): (**a**) illustration of the analyzed cross-section of the functional demonstrator and (**b**) the backscatter electron microscopy of the polished cross-section of the soldered joint. The examples of the pores in the glass solder are highlighted by white arrows.

Structural analysis of the soldered joint of a TiNbTa-ATZ functional demonstrator revealed a homogenous joint gap and pores within the glass solder (Figure 8), as already observed for the soldered cylindrical specimens (Figure 5 and Figure 6). Due to the high quality of the bond, all specimens survived a fatigue test with 10,000-simulated gait cycles. The functional demonstrators showed a brittle fracture behavior, which is illustrated by the moment–rotation curves shown in Appendix B. The maximum extension–flexion moments of the functional demonstrators consisting of TiNbTa-ATZ and Ti-ATZ were 40.7 ± 2.2 Nm (individual values: 42.2 Nm, 42.3 Nm, 37.7 Nm) and 18.4 ± 3.8 Nm (individual values: 17.2 Nm, 14.5 Nm, 23.5 Nm), respectively. In accordance with the shear-induced fracture, all specimens showed a mixed cohesive and adhesive failure.

## 4. Discussion

Multifunctional hybrid materials have been described as reducing the rate of material-related aseptic implant loosening in total joint replacements [13,14,15,16,17,18,19,20,21]. These hybrid materials are composed of an oxide ceramic at the articulating interfaces and a Ti-based material at the bone–implant interface. One feasible technology to combine oxide ceramics with Ti alloys is glass soldering [20,21,66,67]. Here, we investigated the static and fatigue shear strength, the influence of aging, and the cytotoxicity of hybrid material specimens consisting of a slip-casted ATZ and additively manufactured β-type Ti-35Nb-6Ta that were joined by a silica-based glass solder. In addition, the biomechanical performance of the functional demonstrators of a total knee replacement was analyzed under walking cycles, and load-to-failure testing was conducted under an extension–flexion loading.

The static shear strength of the TiNbTa-ATZ hybrid material (26.4 ± 4.2 MPa) did not differ significantly from that of Ti-ATZ (38.2 ± 14.4 MPa), and accelerated aging did not significantly affect the shear strength (see Figure 6a). In addition, all specimens demonstrated sufficient fatigue strength to withstand 10^7^ dynamic shear loading cycles. A comparable study investigating Ti-ZrO_2_ hybrid materials reported a shear strength of 16.8 ± 4.9 MPa [25], which is slightly below the values observed in this present study.

In order to be used as an implant material in cementless total joint replacements, the soldered joint of the hybrid material should not represent a predetermined fracture point. In the case of cementless titanium-based implants, the fixation strength between the bone and the implant surface determines the maximum load-bearing capacity. It has been reported that the bone–implant interface strength ranges from 0.5 MPa to 19.7 MPa [68,69,70,71,72]. In addition, in the standard to evaluate the shear strength of titanium-based plasma-sprayed coatings, 20 MPa has been defined as a minimum requirement [63]. Therefore, according to the measured properties, the investigated hybrid materials had sufficient strength to ensure that they do not form a flaw when used in endoprosthetic implants. However, during functional loading in vivo, the hybrid material is subjected to mixed tensile, shear, and compressive stresses [73]. Investigating the influence of the different stresses occurring simultaneously is complex and requires further studies that go beyond the content of the present study.

The microscopic investigations of the fracture surfaces (Figure 6) revealed that the strength of the hybrid material is determined by the adhesive failure along the interface between the glass solder and Ti-based material, as well as by the cohesive fracture of the glass solder, which is in line with previous observations [21,25,67]. Within the TiNbTa alloys, oxide films (e.g., TiO_2_, Nb_2_O_5_, and Ta_2_O), are formed [74,75], and the reaction of the chemical compounds in the surface layers with the glass solder is crucial in the formation of the material bond [20,21]. Hey et al. [76] described the formation of Ti_5_Si_3_ due to the reaction of SiO_2_ with Ti using a comparable silica-based glass solder. Furthermore, in a study on the diffusion bonding of Al_2_O_3_ and cp-Ti, Travessa et al. [77] described that, at 800 °C, Al_2_O_3_ dissolves in the presence of titanium and further reacts with titanium to form an intermetallic Ti_3_Al compound. In the process, oxygen diffuses into titanium, and Al-rich compounds accumulate at the interface, which was also observed in our study. In contrast, no chemical reaction in the Ti-30Ta and Ti-40Nb with Al_2_O_3_ has been reported [78,79], and also no measurable formation of an interfacial reaction phase of pure Nb with Al_2_O_3_ has been conducted [80]. Therefore, it seems reasonable that the material bond between the glass solder and TiNbTa was formed by the reaction of titanium with SiO_2_ and Al_2_O_3_. Moreover, it has been previously described that the formed oxide layer in the titanium material or the interface between the oxide layer and the bulk material was responsible for the failure of the interfaces between the titanium and glass ceramics [21,67]. This was also shown in our present study by the visible deposition of the TiNbTa or cp-Ti on the ATZ fracture surface. The described chemical reactions should be verified in future research by focusing on the formation of the intermetallic reaction zone.

Transferring the knowledge gained from glass soldering to more complex and larger joining surfaces is crucial for the development of a hybrid material-based endoprosthetic implant. To gain a first experience of the feasibility, we manufactured a simplified functional demonstrator resembling one condyle of the tibiofemoral joint (see Figure 2). As we already observed the pores in the soldered joints of the shear test specimens, we tried to reduce them by modifying the priming of the joining surfaces. To achieve a constant joint thickness, the titanium components were provided with spacers that were 0.1 mm in height. The dimensions of the functional demonstrator are shown in Figure 2 and Appendix A. Despite these efforts, pores were still visible in the joint gaps. Nevertheless, all specimens survived 10,000 walking cycles, and the TiNbTa-ATZ hybrids showed maximum extension–flexion moments of 40.7 ± 2.2 Nm. The rather small standard deviation indicates that the modification of the priming processes had a positive influence on the variations in the mechanical properties.

Given the absence of prior experiences with the investigated hybrid material regarding biomechanical loading scenarios, the walking cycles gave a first impression of the biomechanical performance of the implant demonstrator. We admit that 10,000 cycles are not enough to prove the fatigue strength under physiological loading. For example, the ISO standard 14,243 specifies 5 × 10^6^ load cycles, which correspond to approximately five years of clinical use. In addition to the walking cycles, a subsequent loading to failure was used to determine the maximum extension–flexion moment. Bergmann et al. [81] reported data of an instrumented TKR and defined the EXTREME100 case as the maximum value suitable for studying mechanical safety under severe in vivo conditions. The flexion moments during walking and jogging were 25.9 Nm and 39.8 Nm, respectively; in addition, significantly higher values of 46.1 Nm and 59.1 Nm have been observed during squatting and stair descent, respectively [81]. Another study by Dreyer et al. [82] determined the peak values during various physiological motions in a comparable range (26 to 35 Nm). Considering that the maximum extension–flexion moment of the functional demonstrator was observed for a single condyle and that in vivo loads were measured for a bicondylar TKR, it seems that the bonding strength of the TiNbTa-ATZ hybrid meets the minimum requirement for an endoprosthetic implant. However, as mentioned above, the total knee endoprostheses were subjected to complex loadings by superimposed forces and moments. In addition, the material joint strength should provide a high safety factor that ensures mechanical functionality over a long period. The bonding strength of the complex-shaped hybrid material specimens should therefore be improved, e.g., by realizing a form fit of the ATZ and titanium components.

We observed a difference in the maximum extension–flexion moments of the TiNbTa-ATZ and Ti-ATZ functional demonstrators, although no significant differences were observed during the shear loading tests. For shear loading, the additive manufactured TiNbTa was machined to obtain parallel joining surfaces, which was afterward sandblasted (see Section 2.2). This procedure led to a similar roughness of the different specimens. However, the joining surfaces of the TiNbTa components of the functional demonstrator were not machined, and only sandblasting of the as-printed surface with similar process parameters to the cp-Ti components was used. For this reason, the TiNbTa components possessed a higher roughness than those of cp-Ti. In addition to the chemical bond, mechanical interlocking can also majorly contribute to bonding strength [20], which may have led to the increased joint strength in the rougher TiNbTa demonstrators. However, no study has investigated the influence of the surface roughness of additively manufactured TiNbTa components on bonding strength with a silica-based glass solder so far. Therefore, this might be one factor to improve, i.e., further increasing the bonding strength.

In addition to the mechanical properties, the cytotoxicity of the hybrid materials specimens is relevant for their later application as bone implants. In our present study, the TiNbTa-ATZ specimens did not impair the vitality of the human osteoblasts, whereas Co-28Cr-6Mo decreased the cell proliferation and metabolic activity (see Figure 7). The cytotoxic effect of the released Co- and Cr-ions on human cells has been previously demonstrated in various studies [4,5,6,7,8]. In contrast to Co-28Cr-6Mo, the glass solder and ATZ ceramics are highly biocompatible [26]. In addition, it has been shown that osteoblasts cultured on TiNbTa exhibit a gene differentiation indicating bone formation [47], and Ti/Nb/Ta alloys are highly corrosion-resistant [74,75]. Furthermore, contrary to Ti-6Al-4V, where potentially harmful aluminum and vanadium ions are released [9,10,11,34], niobium and tantalum are highly biocompatible with no cytotoxic effects have been described so far [83]. In line with these previous findings, we demonstrated that the hybrid TiNbTa-ATZ material showed no cytotoxic effects in vitro; however, future studies may need to investigate the ion release in the long term.

Having said the above, this study has some limitations. We observed the pores in the soldered interface that reduce the mechanically loaded cross-section area. Such faults may cause local stress concentrations resulting in unexpected failure. The pores are based on entrapped gas, which might come from the evaporation of the polymer-based carrier suspension of the glass solder paste during firing. Minimizing the pore formation is a critical issue for the manufacturing of reliable bonding with glass solders [24,84]. The development of technological approaches to prevent these pores was beyond the scope of this present study. All the TiNbTa components were heat-treated before soldering to reduce the residual stresses during soldering; however, comprehensive investigation of the influence on the microstructure and mechanical properties was not performed in this study. Furthermore, despite measures (such as PBF-LB/M in an argon atmosphere and soldering in a vacuum) to prevent the oxidation of the TiNbTa component, the influence on the aforementioned properties cannot be completely ruled out. Moreover, oxygen might diffuse from the glass solder into the TiNbTa alloy. The influence of the manufacturing chain on the mechanical properties of the TiNbTa alloy is a part of ongoing studies, and it will be addressed in the future. In addition, the functional demonstrator represents a simplified implant design. Hence, the observations during biomechanical testing need to be verified with a more complex design that is closer to the currently used implants.

Further research should focus on parameter characterization for the bonding strength of TiNbTa-ATZ hybrid materials, e.g., by characterizing the influence of the surface roughness or the chemical composition of the glass solder and joining parameters.

## 5. Conclusions

Aseptic implant loosening of joint endoprostheses is partially affected by the currently used implant materials. This study described the manufacturing of the advanced TiNbTa-ATZ hybrid materials that potentially combine the high wear and corrosion resistance of the ATZ ceramics and enhanced osseoconductivity of TiNbTa alloys. The mechanical characterizations within shear tests and biomechanical loading scenarios that were applied to the functional demonstrators of the femoral component of total knee replacements revealed a sufficient enough mechanical strength to withstand acting loads during physiological motion. Furthermore, in line with the intrinsic properties of the specific materials, the TiNbTa-ATZ hybrid material showed no cytotoxic effect on human osteoblasts. Therefore, our data indicate the potential of hybrid TiNbTa-ATZ implant materials for use in joint endoprostheses.

## Figures and Tables

**Table 1:** Overview of the mechanically tested groups, the used hybrid material, the test specifications, and the measured cross-section at the soldered joint.

Group	Material	Specifications	Cross-Section [mm^2^]
1	TiNbTa-ATZ	Static shear test	279.4 ± 0.1
2	Ti-ATZ	Static shear test	280.0 ± 0.3
3	TiNbTa-ATZ	Accelerated aging followed by static shear test	281.0 ± 1.1
4	Ti-ATZ	Accelerated aging followed by static shear test	280.4 ± 0.4
5	TiNbTa-ATZ	Fatigue shear test	280.3 ± 0.8
6	Ti-ATZ	Fatigue shear test	281.9 ± 1.2

**Figure A1:**
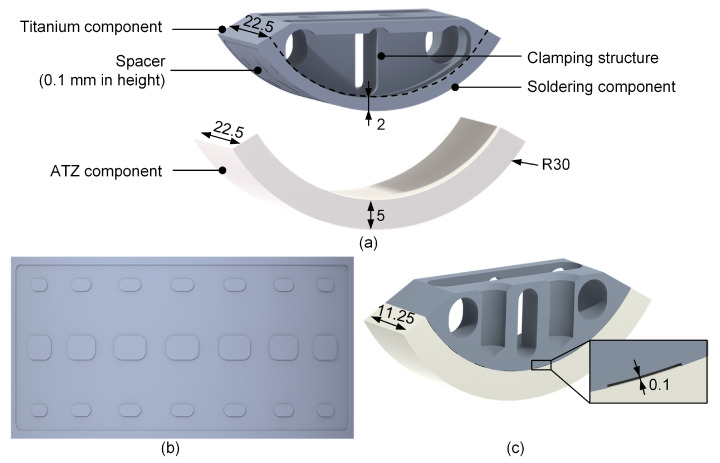
Depiction of the designed hybrid-material-based functional demonstrator of a total knee replacement showing (**a**) an explosion view and the dimensions of the components, (**b**) the designed spacers on the titanium component to obtain a homogenous joint gap, and (**c**) a cut view of the functional demonstrator to highlight the designed joining interface.

**Figure A2:**
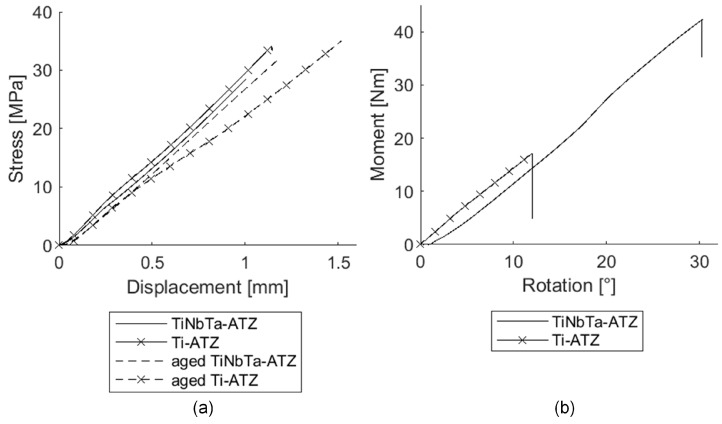
Representative results of the hybrid materials made of an alumina-toughened zirconia (ATZ) ceramic and an additive manufactured Ti-35Nb-6Ta (TiNbTa) or cp-Ti joined with a silica-based glass solder. (**a**) Stress–displacement curves of the static shear tests with the TiNbTa-ATZ (Group 1) and Ti-ATZ (Group 2) specimens, and (**b**) the representative moment–rotation curves of functional demonstrators of a total knee replacement made of TiNbTa-ATZ or Ti-ATZ hybrid material.

## Data Availability

The data presented in this study are available on request from the corresponding authors.
